# Associations of circulating fatty acids with incident coronary heart disease: a prospective study of 89,242 individuals in UK Biobank

**DOI:** 10.1186/s12872-023-03394-6

**Published:** 2023-07-21

**Authors:** Danyao Jin, Eirini Trichia, Nazrul Islam, Sarah Lewington, Ben Lacey

**Affiliations:** 1 Clinical Trial Service Unit & Epidemiological Studies Unit, Nuffield Department of Population Health, University of Oxford, Oxford, UK; 2 MRC Population Health Research Unit, University of Oxford, Oxford, UK; 3 Faculty of Medicine, University of Southampton, Southampton, UK; 4 UK Biobank, Stockport, Greater Manchester, UK

**Keywords:** Fatty acids, Coronary heart disease, Lipids, Nuclear magnetic resonance, UK Biobank

## Abstract

**Background:**

The role of fatty acids in coronary heart disease (CHD) remains uncertain. There is little evidence from large-scale epidemiological studies on the relevance of circulating fatty acids levels to CHD risk. This study aims to examine the independent associations of the major circulating types of fatty acids with CHD risk.

**Methods:**

UK Biobank is a prospective study of adults aged 40–69 in 2006–2010; in 2012–2013, a subset of the participants were resurveyed. Analyses were restricted to 89,242 participants with baseline plasma fatty acids (measured using nuclear magnetic resonance spectroscopy) and without prior CHD. Cox proportional hazards models were used to estimate hazard ratios (HRs) for the associations with incidence CHD, defined as the first-ever myocardial infarction, unstable angina pectoris, coronary-related death, or relevant procedure. And the major types of fatty acids were mutually adjusted to examine the independent associations. Hazard ratios were corrected for regression dilution using the correlation of baseline and resurvey fatty acids measures.

**Results:**

During a median follow-up of 11.8 years, 3,815 incident cases of CHD occurred. Independently of other fatty acids, CHD risk was positively associated with saturated fatty acids (SFA) and monounsaturated fatty acids (MUFA), inversely associated with omega-3 polyunsaturated fatty acids (PUFA), but there was no strong evidence of an association with omega-6 PUFA: HR per standard deviation higher were 1.14 (95% CI, 1.09–1.20), 1.15 (1.10–1.21), 0.91 (0.87–0.94), and 1.04 (0.99–1.09) respectively. Independently of triglycerides and cholesterol, the inverse association with omega-3 PUFA was not materially changed, but the positive associations with SFA and MUFA attenuated to null after adjusting for triglycerides levels.

**Conclusions:**

This large-scale study has quantitated the independent associations of circulating fatty acids with CHD risk. Omega-3 PUFA was inversely related to CHD risk, independently of other fatty acids and major lipid fractions. By contrast, independently of other fatty acids, the positive associations of circulating SFA and MUFA with CHD risk were mostly attributed to their relationship with triglycerides.

**Supplementary Information:**

The online version contains supplementary material available at 10.1186/s12872-023-03394-6.

## Introduction

Dietary guidelines commonly recommend reducing total fat intake, and replacing saturated fat with polyunsaturated fat to lower cardiovascular disease (CVD) risk [1–3]. These guidelines are based largely on evidence from randomized controlled trials of dietary intake of fats,[4, 5] but the effects of the different types of polyunsaturated fats (particularly omega-3 and omega-6), or of replacement of saturated fats with monounsaturated fats, remains unclear [4, 6, 7].

Fat consumption is known to affect circulating levels of fatty acids, which are the main constitutional component of circulating lipid classes and has been shown to modulate lipid metabolism [3, 8, 9]. Although the importance of blood lipids, including low-density-lipoprotein cholesterol and triglyceride levels, to CHD risk is well established, the strength of the associations between circulating fatty acids levels and CHD risk remains unclear. In addition, the relevance of fatty acids levels independently of blood lipids has not been well described [10]. Understanding these associations and the underlying biological mechanisms between circulating fatty acids and CHD risk are important to the development of dietary guidelines, and may inform clinical trials targeting circulating levels of particular fatty acids.

Previous observational studies of the associations of circulating fatty acid levels to CHD risk have tended to be small in size, likely reflecting the challenges of storing and analyzing blood samples at scale, and most lacked repeated measurements of fatty acids making them prone to underestimating the associations due to regression dilution bias [11, 12]. Furthermore, most of these studies failed to account adequately for the high correlations between some fatty acids, which may affect the interpretation of findings [13, 14]. The analyses in the present report use UK Biobank, a large-scale cohort study, to quantify reliably the associations of CHD risk with the major circulating types of fatty acids, independently of each other and major lipid fractions.

## Methods

### Study design and population

UK Biobank is a prospective cohort study of approximately 0.5 million adults in the United Kingdom recruited from 2006 to 2010 [15, 16]. At recruitment, information about sociodemographic factors, lifestyle and health-related characteristics were collected by computer-based questionnaires, and clinical measurements including anthropometrics and blood pressure were made. Blood samples were collected for long-term storage. A subset of 20,346 participants received a resurvey during 2012 to 2013, comprising the full baseline assessment. Ethics approval for the UK Biobank study was given by the National Health Service North West Multicentre Research Ethics Committee.

### Measurement of fatty acids

Plasma fatty acids were assayed by a high-throughput nuclear magnetic resonance (NMR) spectroscopy platform (Nightingale Health, Finland) for 117,980 participants at baseline (a random subset of the initial cohort) and 5,306 participants at resurvey (a random subset of the resurveyed participants) [17–19]. The quantified plasma fatty acids represent a combination of fatty acids in lipid fractions (i.e. triglycerides, phospholipids, or cholesterol esters) and free fatty acids (also called non-esterified fatty acids) [20]. The fatty acid biomarkers included long-chain omega-3 docosahexaenoic acid (DHA), omega-6 linoleic acid, total omega-3 PUFA, total omega-6 PUFA, total PUFA, total MUFA, total SFA, and total fatty acids. Both the concentration of each fatty acid and the corresponding percentage (by weight) of total fatty acids were calculated.

### Ascertainment of incident CHD

Incident CHD was defined as the first-ever myocardial infarction, unstable angina pectoris, or coronary-related death using codes of the 10th edition of the International Classification of Disease (ICD-10), and coronary-related procedures (coronary artery bypass surgery or percutaneous transluminal angioplasty stent placement) by the OPCS Classification of Interventions and Procedures. Incident events were identified from hospital episode statistics (HES) and from the Office for National Statistics (ONS) cause of death data (Table S1) [21].

### Statistical analysis

We excluded participants who aged less than 40 or more than 70 years at baseline, withdrew from the study at the time of analysis, were missing fatty acid biomarkers or key covariates, had outlying values of fatty acids (both outside the range of 4 standard deviations and outside of 0.003% of either side of the distribution), had prior CHD (identified by HES records and baseline self-report) or were taking lipid-lowering medication (e.g. statins) at baseline (Figure S1).

Cox proportional-hazards models, stratified by sex and age (in 5-year groups), were used to derive hazard ratios (HRs) for the associations of fatty acids with incident CHD; HRs are reported per standard deviation higher level of each fatty acid. Models were first adjusted for education, region, Townsend Deprivation Index, [22] smoking, and alcohol intake, and then further adjusted for body-mass index (BMI). HRs were corrected for regression dilution bias (i.e. categorising people by their baseline fatty acid and estimating the long-term average mean fatty acid in each category using the correlation between re-survey and baseline measurements), and are therefore described as associations of usual fatty acids with CHD risk [11, 12]. ‘Usual’ levels in the plot were estimated from the mean value at resurvey within each baseline defined group, representing an unbiased estimate of the long-term average level in each baseline-defined group. The standard deviation (SD) of the usual value was obtained by multiplying the baseline SD by the square root of the regression dilution ratio. Confidence intervals (CIs) were calculated using the variance of the log risk, which appropriately attributes variance to all groups, including the reference [23, 24].

To examine the independent association of each fatty acid, other fatty acids were progressively added to the model. To explore whether the association of the fatty acid with CHD risk was independent of lipids, we further adjusted for plasma cholesterol and for plasma triglyceride level. The Change in the log-likelihood (LR) χ^2^ statistic with and without the fatty acid is a measure of extent of variance explained by the fatty acid in addition to the other variables in the model. This statistic provides a significance test for the improvement in fit from including the fatty acid term.

Sensitivity analyses were conducted by excluding CHD events in the first two years of follow-up (to assess for potential reverse causality), and by further adjusting for other potential confounders. Supplementary analyses on the ratio biomarkers (i.e. the percentage of different types of fatty acid to total fatty acids) were also assessed. All analyses were conducted with SAS version 9.4 and all figures were generated in R version 4.0.1.

## Results

After exclusions, 89,242 participants at baseline were included in the main analyses (Figure S1). During a median follow-up of 11.8 years, there were 3,815 incident CHD events, of which 488 events were CHD death (Table S2). On average, participants at baseline who developed incident CHD were slightly older, more likely to be male and current smokers, and have lower education and higher levels of adiposity measures and systolic blood pressure, and higher percentage of diabetes (Table 1). Other baseline comparisons were provided in Table S3.

**Table 1:** Baseline characteristics by incident coronary heart disease

	Incident coronary heart disease		All
	No	Yes	
**No. of participants**	85,427	3,815	89,242
**Age, sex and socioeconomic factors**			
Baseline age, years	55.0 (8.0)	59.2 (7.1)	55.2 (8.0)
Male, %	41.8	64.5	42.8
White, %	95.0	95.4	95.0
University education, %	40.8	31.6	40.4
Townsend Deprivation Index*	-1.4 (3.0)	-1.2 (3.2)	-1.4 (3.0)
**Lifestyle factors**			
Current smoker, %	10.2	16.5	10.5
Current regular alcohol drinker, %	70.7	68.4	70.6
**Anthropometry**			
Body Mass Index, kg/m^2^	26.9 (4.6)	27.9 (4.6)	27.0 (4.6)
Waist circumference, cm	88.5 (12.9)	93.9 (12.5)	88.7 (12.9)
Waist to hip ratio	0.86 (0.09)	0.91 (0.08)	0.86 (0.09)
**Lipids measured by clinical chemistry** ^†^			
LDL cholesterol, mmol/l	3.7 (0.8)	3.9 (0.8)	3.7 (0.8)
HDL cholesterol, mmol/l	1.5 (0.4)	1.3 (0.4)	1.5 (0.4)
Total triglycerides, mmol/l	1.7 (1.0)	2.0 (1.1)	1.7 (1.0)
**Blood pressure and diabetes**			
Systolic blood pressure, mmHg	136.3 (18.4)	144.8 (19.0)	136.7 (18.5)
Diastolic blood pressure, mmHg	82.1 (10.2)	85.1 (10.54)	82.2 (10.2)
Baseline diabetes, %	2.0	4.4	2.1

Baseline characteristics of those with and without incident coronary heart disease during follow up among 89,242 participants (exclusions as in Figure S1). Continuous variables are presented as mean (standard deviation) and categorical variables are presented as column percentages. *Area-level measure of material deprivation, higher scores represent higher levels of deprivation. †Measured directly. LDL = low-density lipoproteins; HDL = high-density lipoproteins.

At baseline, the concentration of SFA was highly correlated with the concentration of MUFA (Spearman correlation, r = 0.93). The concentration of omega-6 PUFA was moderately correlated with the concentration of SFA (r = 0.75) and of MUFA (r = 0.73), while omega-3 PUFA had lower correlations with the other types of fatty acid (r = 0.38 to 0.47) (Table S4). SFA and MUFA had high correlations with total triglycerides (0.87 and 0.93, respectively), and omega-6 PUFA had high correlation with non-HDL-C (0.82). The correlations between omega-3 PUFA and all lipid biomarkers were lower (0.16 to 0.40) (Table S5).

A total of 1,283 participants had both baseline and resurveyed NMR-derived fatty acids measures. Following the same exclusion criteria as baseline participants, 1,053 participants were included for the analyses (on average 4.2 years after the baseline assessment). The characteristics of resurveyed participants were broadly similar to those included in the main analyses, except for a slightly higher level of education and lower percentage of smokers among those resurveyed (Table S6). The mean concentrations of fatty acid measures were also similar at baseline and resurvey (Table S7), and the correlations of these measures, which represented the regression dilution ratios, ranged from 0.51 to 0.62 (Table S8).

**Fig. 1:**
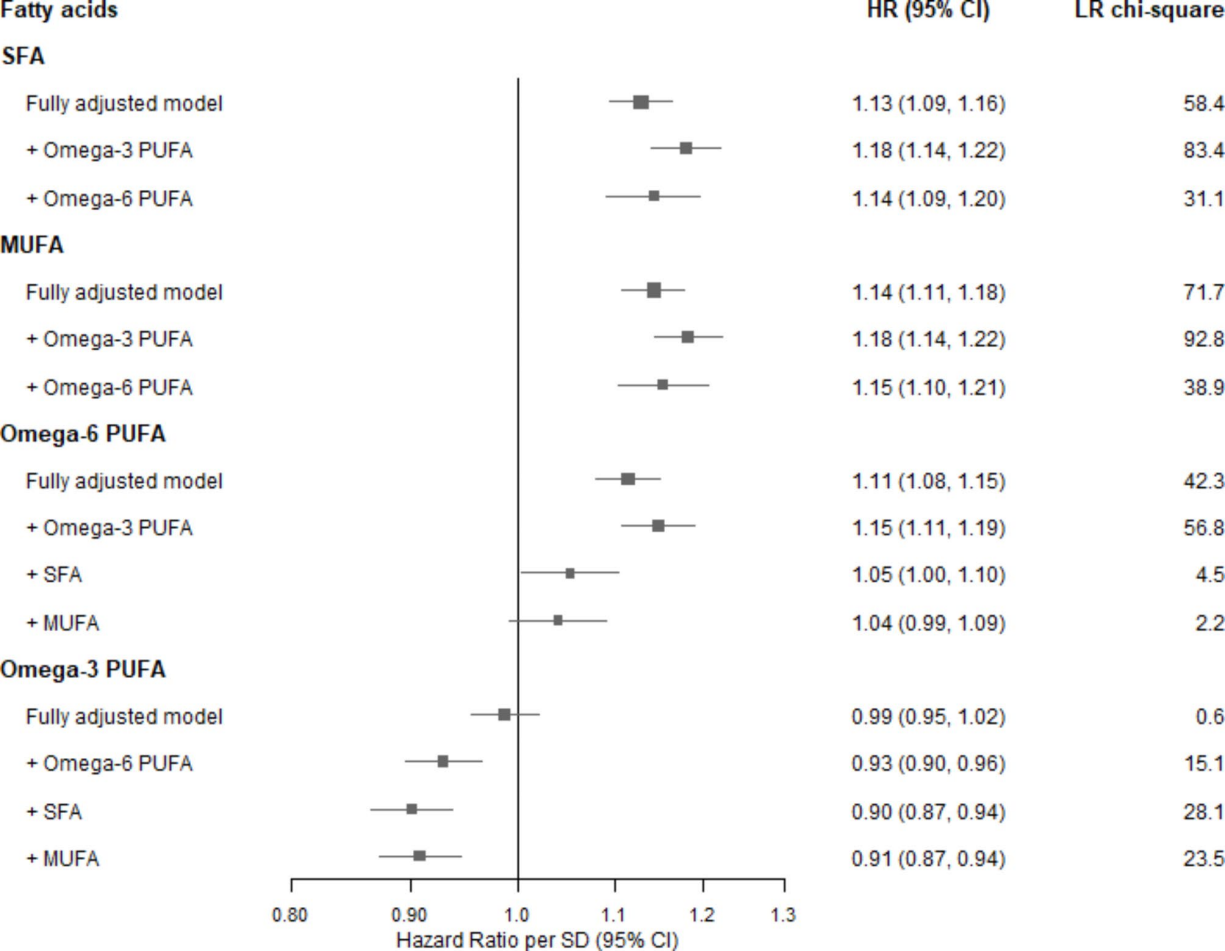
Risk of coronary heart disease by usual fatty acids concentration, with progressively adjustment for other fatty acids. Hazard ratio (HR) per usual SD higher level of each mean fatty acid ratio among 89,242 participants (usual SD was estimated by 1,053 resurveyed participants). HRs were calculated by Cox proportional-hazards models with stratification by age and sex, and adjustment for ethnicity, education, region, Townsend Deprivation Index, smoking, alcohol, and body-mass index; and further adjustment for each fatty acid concentration progressively. Likelihood ratio (LR) χ2 improvement with the addition of the given factors to the model with stated adjustments. SFA = Saturated fatty acids; MUFA = Monounsaturated fatty acids; PUFA = Polyunsaturated fatty acids

**Fig. 2:**
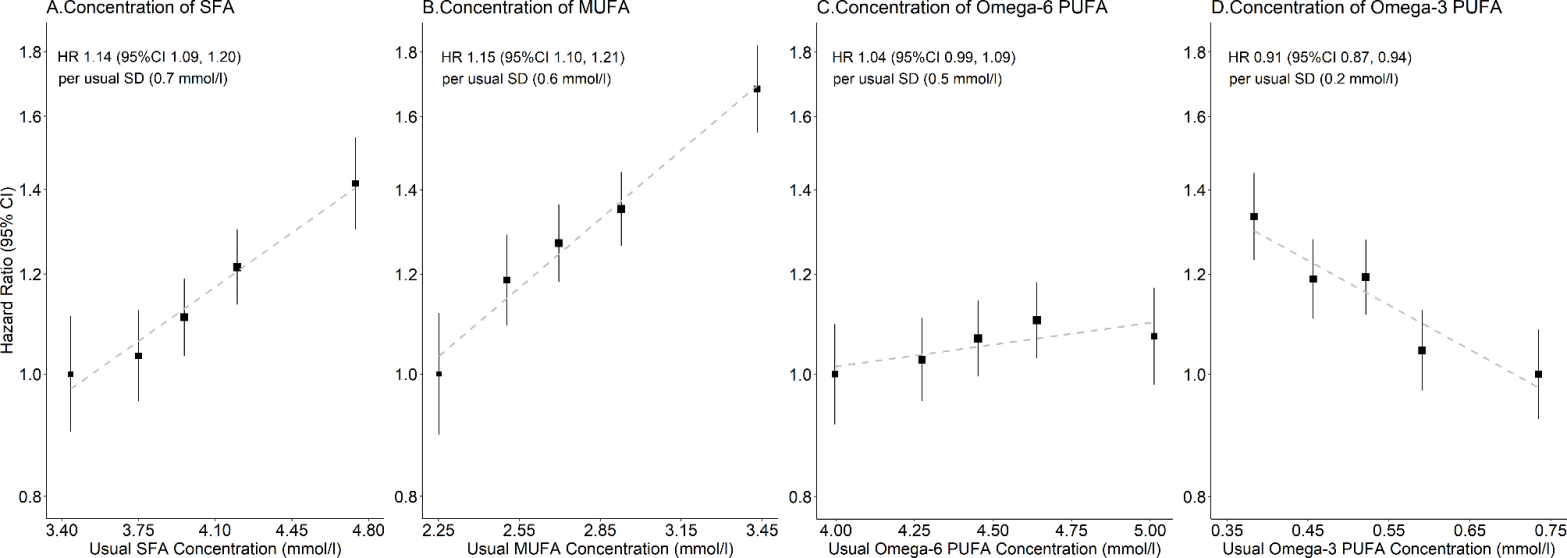
Fatty acids concentration vs. coronary heart disease risk, with adjustment for other fatty acids. Hazard ratio (HR) per usual SD higher level of each fatty acid concentration among 89,242 participants (usual SD was estimated by 1,053 resurveyed participants). HRs were calculated by Cox proportional-hazards models, stratified by age and sex, and adjusted for ethnicity, education, region, Townsend Deprivation Index, smoking, alcohol, body-mass index, and mutual adjusted for other fatty acids (as last row of each type of fatty acid in Fig. 1). Area of the square is inversely proportional to the variance of the category-specific log risk. SFA = Saturated fatty acids; MUFA = Monounsaturated fatty acids; PUFA = Polyunsaturated fatty acids

There were linear associations of usual levels of all the fatty acids with incident CHD (Figure S2). Both circulating SFA and MUFA had strong positive associations with incident CHD in fully adjusted models (HR per usual SD, 1.13 [95% CI 1.09–1.16] and 1.14 [1.11–1.18], respectively: Table 2), although the adjustment of BMI slightly attenuated the associations (Table S9). Furthermore, the positive linear associations of SFA and MUFA remained largely unchanged when independently of other types of fatty acids (omega-3 and omega-6 PUFA) (Figs. 1 and 2) (the associations of SFA and MUFA were not further adjusted for each other because of the very high correlation between these variables).

**Table 2:** Association of fatty acids concentration with coronary heart disease risk

Fatty acids concentration	Fatty acids (mmol/L)		A. Adjusted for age and sex		B. Further adjusted for other confounders		C. Further mutually adjusted for other fatty acids	
	Baseline mean	Usual SD	HR (95% CI)*	LR χ^2†^	HR (95% CI)*	LR χ^2†^	HR (95% CI)*	LR χ^2†^
**SFA**	4.1	0.7	1.17 (1.14,1.21)	107.9	1.13 (1.09,1.16)	58.4	1.14 (1.09,1.20)	31.1
**MUFA**	2.8	0.6	1.21 (1.18,1.24)	159.2	1.14 (1.11,1.18)	71.7	1.15 (1.10,1.21)	38.9
**PUFA**								
**Omega-6 PUFA**	4.5	0.5	1.12 (1.08,1.15)	44.3	1.11 (1.08,1.15)	42.3	1.04 (0.99,1.09)	2.2
**Linoleic acids**	3.5	0.5	1.11 (1.07,1.14)	39.8	1.11 (1.08,1.15)	42.7	1.03 (0.99,1.08)	1.9
**Omega-3 PUFA**	0.5	0.2	0.94 (0.91,0.98)	11.3	0.99 (0.95,1.02)	0.6	0.91 (0.87,0.94)	23.5
**DHA**	0.2	0.1	0.87 (0.84,0.90)	64.3	0.94 (0.91,0.98)	10.6	0.92 (0.88,0.95)	19.9

Hazard ratio (HR) per usual SD higher level of each mean fatty acid concentration among 89,242 participants (usual SD was estimated by 1,053 resurveyed participants). *HRs were calculated by Cox proportional-hazards models with: (A) stratification by age and sex; (B) stratification by age and sex, and adjusted for ethnicity, education, region, Townsend Deprivation Index, body-mass index, smoking, and alcohol; (C) model B with further mutually adjusted for other fatty acids (as last row of each type of fatty acids in Fig. 1). ^†^Likelihood ratio (LR) χ2 improvement with the addition of the given factors to the model with stated adjustments. SFA = Saturated fatty acids; MUFA = Monounsaturated fatty acids; PUFA = Polyunsaturated fatty acids; DHA = Docosahexaenoic acid.

There were also positive associations of omega-6 PUFA and its major subtype, linoleic acid, with CHD risk, which was little altered by the adjustment of BMI (1.11 [1.08–1.15] for both) (Table 2, Table S9). However, the HR of omega-6 PUFA in the fully adjusted model was substantially attenuated to 1.05 (1.00-1.10) when further adjusting for SFA, and to 1.04 (0.99–1.09) when further adjusting for both SFA and MUFA (Fig. 1); the large reduction (92%) in the LR χ^2^ statistic (from 56.8 to 4.5) after further adjustment for SFA and MUFA indicates the positive association of omega-6 PUFA with CHD risk may be largely accounted by the effects from these other fatty acids. The independent association of linoleic acid given other fatty acids followed a similar pattern with the independent association of omega-6 PUFA (Table 2, Figure S3).

In terms of omega-3 PUFA, there was no evidence of an association between the overall concentration with CHD risk in the fully adjusted model (0.99 [0.95–1.02]), and a slight inverse association (0.94 [0.91–0.98]) of DHA, a subtype of omega-3 PUFA (Table 2). However, the associations of omega-3 PUFA and DHA changed from null to significantly inverse in all models with progressive adjustments of other fatty acids (0.91 [0.87–0.94] and 0.92 [0.88, 0.95], respectively: Table 2; Fig. 1). The plot of the independent associations of omega-3 PUFA and DHA given other fatty acids are shown in Fig. 2 and Figure S3.

**Fig. 3:**
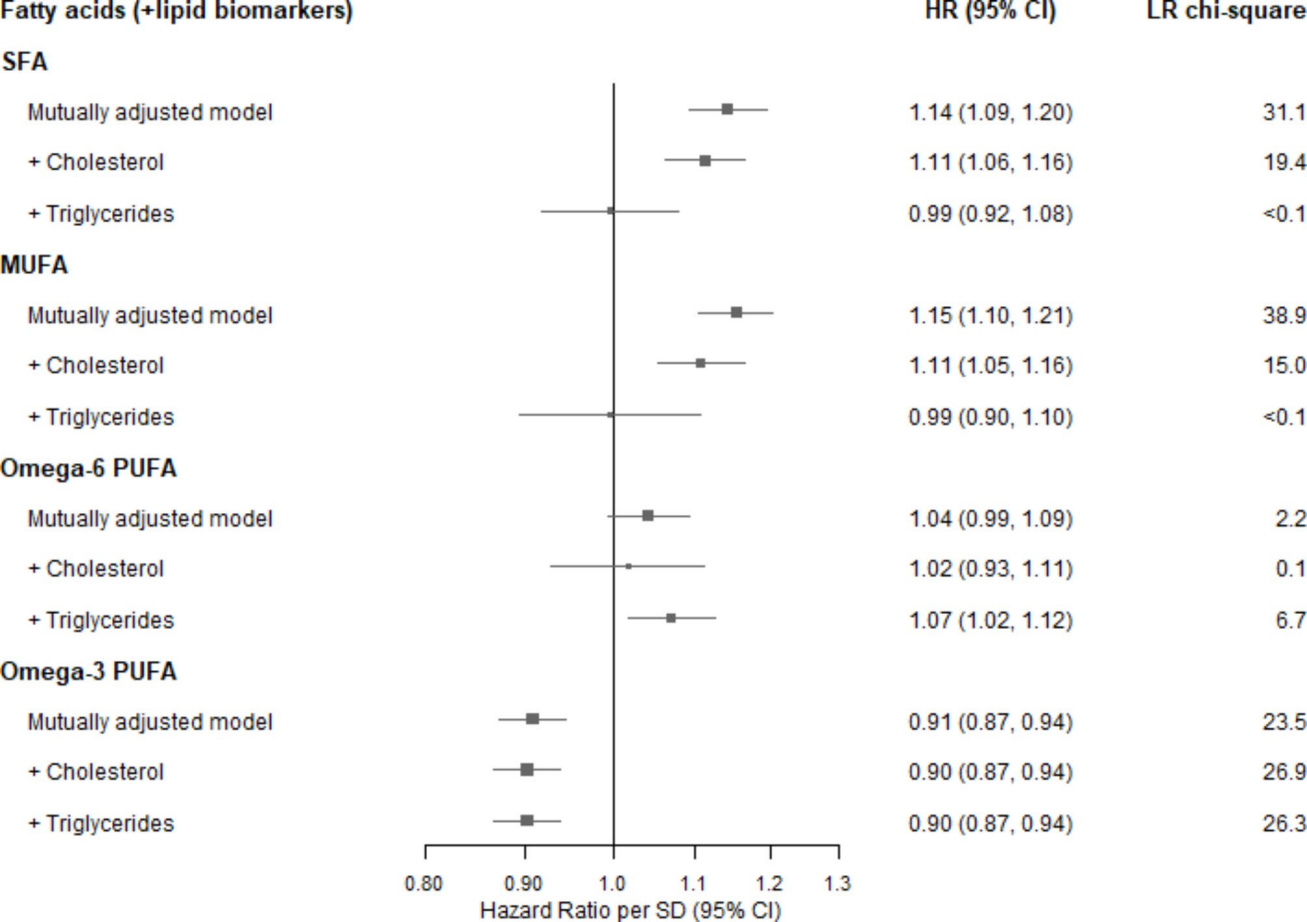
Risk of coronary heart disease by usual fatty acids concentration, with further adjustment for lipids. Hazard ratio (HR) per usual SD higher level of each mean fatty acids concentration among 89,242 participants (usual SD were estimated by 1,053 resurveyed participants). The mutual adjusted model represented Cox proportional-hazards model with stratification by age and sex, and adjustment for ethnicity, education, region, Townsend Deprivation Index, smoking, alcohol, and body-mass index, and mutual adjusted for other fatty acids (as last row of each type of fatty acids in Fig. 1), and further adjustment for non-HDL-cholesterol and HDL-cholesterol, or further adjustment for total triglycerides. Likelihood ratio (LR) χ2 improvement with the addition of the given factors to the model with stated adjustments. SFA = Saturated fatty acids; MUFA = Monounsaturated fatty acids; PUFA = Polyunsaturated fatty acids

The changes of HRs and LR χ^2^ statistics for fatty acids concentrations after further adjusting for cholesterol or triglycerides are shown in Fig. 3. The associations of SFA and MUFA concentrations with CHD risk were attenuated slightly after adjusting for cholesterol levels (HR per SD, 1.11 [1.06–1.18] and 1.11 [1.05–1.16], respectively), but were attenuated to null after adjusting for triglycerides (0.99 [0.92–1.08] and 0.99 [0.90–1.10], respectively). In contrast, there was no evidence that the independent association of omega-6 PUFA and omega-3 PUFA concentrations were altered following further adjustment.

The percentage composition of each fatty acid to total fatty acids with incident CHD was investigated in Table 3 as a complementary analysis. The associations of these percentage measures, including SFA, MUFA and omega-3 PUFA, were similar to the results of concentration biomarkers independently of other fatty acids (Figure S4). The ratios of SFA and MUFA to total fatty acids remained associated with increased CHD risk (1.06 [1.03–1.09] and 1.16 [1.12–1.20], respectively), and higher percentage of omega-3 PUFA were associated with a decreased risk (0.92 [0.89–0.95]). However, in contrast to the results of concentration biomarkers, an inverse association of omega-6 PUFA was observed when expressed as ratio biomarker relative to total fatty acids (0.91 [0.88–0.94]) (Table 3). The percentage composition of each fatty acid independently of cholesterol or triglycerides are shown in Figure S5. Further adjustment of triglycerides attenuated the associations of both SFA and omega-6 PUFA compositions to null, and also modestly attenuated the ratio biomarker of MUFA. Similar to the analyses using concentration levels, the inverse association of omega-3 PUFA to total fatty acids remained unchanged after adjustment of cholesterol or triglycerides.

**Table 3:** Association of fatty acids ratios with coronary heart disease risk

Fatty acids ratio, relative to total fatty acids	Fatty acids ratio (%)		A. Adjusted for age and sex		B. Further adjusted for other confounders	
	Baseline mean	Usual SD	HR (95% CI)*	LR χ^2†^	HR (95% CI)*	LR χ^2†^
**SFA (%)**	34.0	1.2	1.10 (1.07,1.13)	38.9	1.06 (1.03,1.09)	12.7
**MUFA (%)**	23.3	2.0	1.27 (1.23,1.31)	233.0	1.16 (1.12,1.20)	76.3
**PUFA (%)**						
**Omega-6 PUFA (%)**	38.4	2.7	0.85 (0.83,0.88)	107.9	0.91 (0.88,0.94)	35.1
**Linoleic acids (%)**	29.6	2.4	0.89 (0.87,0.92)	53.1	0.96 (0.93,0.99)	7.0
**Omega-3 PUFA (%)**	4.3	1.2	0.85 (0.82,0.88)	91.7	0.92 (0.89,0.95)	26.5
**DHA (%)**	2.0	0.5	0.80 (0.77,0.82)	184.7	0.88 (0.85,0.91)	57.2

Hazard ratio (HR) per usual SD higher level of each mean fatty acids ratio among 89,242 participants (usual SD was estimated by 1,053 resurveyed participants). *HRs were calculated by Cox proportional-hazards models with: (A) stratification by age and sex; (B) stratification by age and sex, and adjusted for ethnicity, education, region, Townsend Deprivation Index, body-mass index, smoking, and alcohol. ^†^Likelihood ratio (LR) χ2 improvement with the addition of the given factors to the model with stated adjustments. SFA = Saturated fatty acids; MUFA = Monounsaturated fatty acids; PUFA = Polyunsaturated fatty acids; DHA = Docosahexaenoic acid.

In sensitivity analyses, exclusion of the first two years of follow-up did not materially alter the main associations (either for the concentration levels independently of other fatty acids or for the percentage composition levels), and neither did further adjustment of other potential confounders, including fasting time and dietary habits (Table S9). Analyses of the associations of fatty acid concentrations with CHD risk among those taking statins were appreciably different to those that excluded statin users, especially for SFA and MUFA (Figure S6).

## Discussion

In this large-scale prospective study, there was strong evidence of positive associations of circulating SFA and MUFA with CHD risk, independently of other fatty acids, and inverse associations of omega-3 PUFA (and DHA). The association with omega-3 PUFA was independent of both triglyceride and cholesterol levels, while the associations of SFA and MUFA were attenuated following adjustment for circulating triglyceride levels. Omega-6 PUFA (and linoleic acid) showed an inverse association with CHD risk when measured as the percentage in total fatty acids, but little evidence of an association in concentration level independently of other fatty acids. This suggests that although the associations of SFA and MUFA are mostly attributed to their relationship with triglycerides, and there are likely to be alternative mechanisms other than lipids lowering by which omega-3 PUFA is related to CHD risk.

This is one of the largest studies to date to quantify the associations of circulating fatty acids with CHD risk, including corrections for regression dilution bias, and assessing the strength of these associations independently of major lipid fractions. Many previous observational studies have assessed the associations of circulating fatty acids with CHD risk expressed as ratios relative to total fatty acids [14, 25, 26]. However, it is challenging to infer the independent associations of fatty acids with CHD risk from such analyses, as it requires simultaneous consideration of the associations of both the numerator and the denominator of the ratio. For example, in the present report we found omega-6 PUFA was unrelated to CHD risk independently of other fatty acids, but there was an inverse association with the ratio of omega-6 PUFA to total fatty acid. This finding may well be driven by the association of CHD risk with total fatty acid (largely composed of SFA and MUFA, which have a positive association with CHD risk), rather than any independent effect of omega-6 PUFA on CHD risk itself. However, both concentration- and percentage-based biomarkers have limitations in exploring the independent effect of fatty acids, and further understanding of the mechanism of fatty acids, especially for omega-6 PUFA, should consider the findings from both types of measurements, and triangulate the results from different study designs, including observational studies, trials, and Mendelian randomization.

Our observation that both plasma SFA and MUFA were associated with higher CHD risk is consistent with a number of previous studies [13, 27]. However, a recent large meta-analysis of observational studies described a positive association of MUFA with CHD risk, but null association of SFA after adjustment for other fatty acids [13]. The present report did not adjust the associations of SFA for MUFA, as there is evidence that level of MUFA may be the potential mediator for the association between SFA and CHD risk, and this may explain the discrepancy in the findings; MUFAs constitute the major fatty acids stored in adipose tissue, and circulating MUFA is largely generated from desaturation of SFA [20, 28].

In the present report, the weak positive associations of circulating omega-6 PUFA and linoleic acid with CHD risk were attenuated to the null after further controlling for other fatty acids, indicating that the association may be due to its correlation with SFA and MUFA. Similarly, a pooled analysis of six UK-based studies assessing plasma linoleic acid showed no evidence of association with CHD risk with and without adjusting for other fatty acids [13]. Meta-analyses of RCTs and prospective cohort studies on replacing SFA dietary intake with PUFA have shown a CHD risk reduction [3–5, 29, 30]. However, such studies which assessing total PUFA have not been able to disaggregate the effects of changes in the different types of PUFA. Omega-6 linoleic acid is the most abundant dietary PUFA and increases in total dietary PUFA are likely to have resulted in high intake of omega-6 linoleic acid, but in theoretically, a concurrent increase in omega-3 may existed and complicate the interpretation of these findings.

Our study showed that circulating level of omega-3 PUFA was independently associated with lower CHD risk, which is consistent with numerous previous observational studies assessing independent risk of plasma fatty acids [13, 14]. Further adjustment of health lifestyle-related factors, such as dietary habit, did not materially change the protective association in circulating level. Omega-3 PUFA has also long been hypothesized at the potential mediator of the inverse association of dietary fish intake with lower CHD risk [31, 32]. Randomized controlled trials on omega-3 PUFA supplementation, however, have not found consistent conclusions on the protective effect of CHD [33–40]. Except for the concerns on patient selection criteria, the duration of treatment and the choice of placebo, the inconsistent conclusions also raised the hypothesis that the formulation of omega-3 PUFA supplementation (Eicosapentaenoic acid [EPA] plus DHA versus pure EPA) may be the key aspect of the effects to cardiovascular event [10, 41]. Circulating levels of EPA and DHA are strongly influenced by dietary intake, and our results showed that higher circulating DHA was associated with decreased CHD risk in similar degree as omega-3 PUFA, which may not support this new hypothesis. RESPECT-EPA trial are ongoing to provide further evidence [42].

Fatty acids are the main constitutional component of lipid classes,[8] and are known to modulate circulating lipids,[3] but current evidence is still unclear how much cholesterol and triglycerides contribute to the relevance of fatty acids to CHD risk. A recent Mendelian randomization (MR) analysis did not support a protective role of circulating PUFA with CVD risk after accounting for LDL-C, but the limitation of MR analysis made it difficult to separate the genetic determinate of PUFA from other dietary changes, and it is also difficult to avoid the bias from horizontal pleiotropy via lipoprotein-related traits [43]. On the other hand, it has been suggested in other study that mechanisms other than lipid-lowering may account for the association between omega-3 PUFA and CHD risk [10]. Our findings showed that further adjustment for triglycerides or cholesterols did not attenuate the inverse association with omega-3 PUFA, indicating that mechanisms other than lipid-lowering may be relevant, which was consistent with the pathophysiologic hypothesis that a combination of various mechanisms contribute to the cardiovascular protection associated with omega-3 PUFA, including anti-inflammation, anti-thrombosis, plaque and membrane stabilization [10, 44]. A recent review of the cardiovascular impact of nutritional supplementation with omega-3 fatty acid also concluded that omega-3 may have beneficial effects other than through triglyceride lowering [10].

### Clinical perspectives

Circulating levels of fatty acids, affected by fat and carbohydrate consumption, are one of the main consititutional components of circulating lipids. Our results indicated the associations of fatty acids with CHD risk, independently of each other and major lipid fractions. Understanding these associations and the underlying biological mechanisms between circulating fatty acids and CHD risk are important to the development of dietary guidelines, and may inform clinical trials targeting circulating levels of particular fatty acids (including estimates of the epidemiologically-expected effect on disease risk of the different fatty acids, and the presence of linear associations throughout the fatty acid ranges examined) to better understand the atherosclerosis mechanism and the threshold of the associations. Furthermore, the results showed strong and robust associations with some types of fatty acids, which are likely to inform the development of risk prediction models to identify those at high risk.

### Strengths and limitations

This study has a number of key strengths, including the large sample size, long follow-up, and reliable ascertainment of CHD events. It is the first large-scale observational study, to our knowledge, to assess the risk of each type of circulating fatty acid independently of major lipid fractions. The resurvey in the subset of study population allowed us to correct for regression dilution, enabling estimates of associations with long-term average levels of fatty acids, which was also not assessed before. Furthermore, the baseline survey collected information on a wide range of factors to allow adjustment for major potential confounders.

Despite this, we cannot exclude the potential for residual confounding or reverse causality in observational studies, and the NMR platform did not include more subtypes of circulating fatty acids. In addition, blood samples in UK biobank were taken in the non-fasting state, which may affect the stability of the measurements. However, recent study found that fasting duration only account for a small proportion of variation in plasma fatty acids concentration[45], and the unchanged results after further adjusting for fasting time and dietary habits in our sensitivity analyses also proved the limited impact of postprandial states on our conclusion. Future analyses should further assess the causality of these associations, including using Mendelian randomization, and explore the relevance of other fatty acid subtypes that are currently unmeasured in the cohort, such as EPA. Metabolomics data will also become available on the whole cohort in the near future and this will increase the precision of the estimated effects in this study, and permit exploration of effect modification of these associations by important characteristics.

## Conclusion

This study quantifies the independent associations of circulating fatty acids with CHD risk. The findings suggest positive associations of circulating SFA and MUFA, inverse association of omega-3 PUFA, and no evidence of association of omega-6 PUFA with CHD risk. Although the associations of SFA and MUFA were mostly attributed to their relationship with triglycerides levels, the study indicates the inverse association with omega-3 PUFA is unlikely to be mediated by major lipid fractions.

## Electronic supplementary material

Below is the link to the electronic supplementary material.

Additional File 1: Associations of circulating fatty acids with incident coronary heart disease: a prospective study of 89,242 individuals in UK Biobank

## Data Availability

Data from the UK Biobank are available to researchers after registration at the UK Biobank server. The data cleaning and coding used to generate the findings of this study are available from the corresponding author on reasonable request.

## List of Abbreviations

BMI: Body mass index
CHD: Coronary heart disease
DHA: Docosahexaenoic acid
HDL: High-density lipoproteins
HR: Hazard ratio
LDL: Low-density lipoprotein
LR: Likelihood ratio
MUFA: Monounsaturated fatty acids
NMR: Nuclear magnetic resonance
PUFA: Polyunsaturated fatty acids
SFA: Saturated fatty acids
