# Supplementary material for: Associations of circulating fatty acids with incident coronary heart disease: a prospective study of 89,242 individuals in UK Biobank
Source: BMC Cardiovasc Disord. 2023 Jul 21;23:365. doi: 10.1186/s12872-023-03394-6 (PMC10362581; doi:10.1186/s12872-023-03394-6)
Supplement: Supplementary file 1 — Additional File 1: Associations of circulating fatty acids with incident coronary heart disease: a prospective study of 89,242 individuals in UK Biobank [file 12872_2023_3394_MOESM1_ESM.docx]

**Associations of circulating fatty acids with incident coronary heart disease: a prospective study of 89,242 individuals in UK Biobank**

Danyao Jin, MSc^*^; Eirini Trichia, PhD^*^; Nazrul Islam, PhD;

Sarah Lewington, DPhil^+^; Ben Lacey, DPhil^+^

^*^ Joint first authors; ^+^ Joint senior authors

**Supplementary Appendix, Table of Contents**

[**Table S1 ICD-10 and operation code of coronary heart disease** 2](#_Toc137214420)

[**Figure S1 Flowchart of exclusion criteria for the study population** 4](#_Toc137214421)

[**Table S2 Major components of incident coronary heart disease** 5](#_Toc137214423)

[**Table S3 Baseline medications and dietary habits by incident coronary heart disease** 6](#_Toc137214424)

[**Table S4 Correlations of baseline fatty acids concentration** 8](#_Toc137214425)

[**Table S5 Correlations of baseline fatty acids concentration and lipid-related biomarkers** 9](#_Toc137214426)

[**Table S6 Baseline characteristics of the resurveyed population** 10](#_Toc137214427)

[**Table S7 Fatty acids biomarkers at baseline versus resurvey among the resurveyed population** 11](#_Toc137214428)

[**Table S8 Regression dilution ratios of fatty acids biomarkers** 12](#_Toc137214429)

[**Table S9 Association of fatty acids concentration with coronary heart disease** 13](#_Toc137214430)

[**Figure S2 Fatty acids concentration vs coronary heart disease risk in minimally adjusted model** 14](#_Toc137214431)

[**Figure S3 Subtype of polyunsaturated fatty acids concentration vs coronary heart disease risk, given other fatty acids** 15](#_Toc137214432)

[**Table S10 Sensitivity analyses of fatty acids and coronary heart disease incidence** 16](#_Toc137214433)

[**Figure S4 Fatty acids ratio vs coronary heart disease risk in fully adjusted model** 17](#_Toc137214434)

[**Figure S5 Risk of coronary heart disease by usual fatty acids ratio, with further adjustment for lipids** 18](#_Toc137214435)

[**Figure S6 Fatty acids concentration vs coronary heart disease risk among participants taking versus not taking statin at baseline, given other fatty acids** 19](#_Toc137214436)

# **Table S1** **ICD-10 and operation code of coronary heart disease**

| ICD/OPCS category | Disease category | Code definition |
| --- | --- | --- |
| I20 | Angina pectoris | I20.0* Unstable angina I20.1 Angina pectoris with documented spasm I20.8 Other forms of angina pectoris I20.9 Angina pectoris, unspecified angina |
| I21 | Acute myocardial infarction | I21.0* Acute transmural myocardial infarction of anterior wall I21.1* Acute transmural myocardial infarction of inferior wall I21.2* Acute transmural myocardial infarction of other sites I21.3* Acute transmural myocardial infarction of unspecified site I21.4* Acute subendocardial myocardial infarction I21.9* Acute myocardial infarction, unspecified |
| I22 | Subsequent myocardial infarction | I22.0* Subsequent myocardial infarction of anterior wall I22.1* Subsequent myocardial infarction of inferior wall I22.8* Subsequent myocardial infarction of other sites I22.9* Subsequent myocardial infarction of unspecified site |
| I23 | Certain current complications following acute myocardial infarction | I23.0* Haemopericardium as current complication following acute myocardial infarction; I23.1* Atrial septal defect as current complication following acute myocardial infarction; I23.2* Ventricular septal defect as current complication following acute myocardial infarction; I23.3* Rupture of cardiac wall without haemopericardium as current complication following acute myocardial infarction;  I23.4* Rupture of chordae tendineae as current complication following acute myocardial infarction  I23.5* Rupture of papillary muscle as current complication following acute myocardial infarction I23.6* Thrombosis of atrium, auricular appendage, and ventricle as current complications following acute myocardial infarction; I23.8* Other current complications following acute myocardial infarction |
| I24 | Other acute ischaemic heart diseases | I24.0 Coronary thrombosis not resulting in myocardial infarction  I24.1* Dressler’s syndrome  I24.8* Other forms of acute ischaemic heart disease  I24.9* Acute ischaemic heart disease, unspecified (excl. ischaemic heart disease (chronic) NOS) |
| I25 | Chronic ischaemic heart disease | I25.0 Atherosclerotic cardiovascular disease, so described  I25.1* Atherosclerotic heart disease  I25.2* Old myocardial infarction  I25.3 Aneurysm of heart  I25.4 Coronary artery aneurysm  I25.5* Ischaemic cardiomyopathy  I25.6* Silent myocardial ischaemia I25.8* Other forms of chronic ischaemic heart disease - Any condition in I21-I22 and I24.- specified as chronic I25.9* Chronic ischaemic heart disease, unspecified -Ischaemic heart disease (chronic) NOS |
| 410 | Acute myocardial infarction | 410.1 Acute myocardial infarction of other anterior wall, episode of care unspecified  410.2 Acute myocardial infarction of inferolateral wall, initial episode of care  410.3 Acute myocardial infarction of inferoposterior wall, episode of care unspecified  410.4 Acute myocardial infarction of other inferior wall, initial episode of care  410.6 True posterior wall infarction, initial episode of care  410.9 Acute myocardial infarction |
| 411 | Other acute and subacute forms of ischaemic heart disease | 411.1 Intermediate coronary syndrome  411.8 Other acute and subacute forms of ischemic heart disease  411.9 Other acute and subacute forms of ischaemic heart disease |
| 412 | Old myocardial infarction | 412.9 Old myocardial infarction |
| 413 | Angina pectoris | 413.0 Angina decubitus  413.1 Prinzmetal angina  413.9 Angina pectoris |
| 414 | Other forms of chronic ischaemic heart disease | 414.0 Coronary atherosclerosis  414.1 Aneurysm of heart  414.8 Other specified forms of chronic ischaemic heart  Diseases  414.9 Chronic ischaemic heart disease, unspecified |
| K40* |  | Saphenous vein graft replacement of coronary artery |
| K41* |  | Other autograft replacement of coronary artery |
| K42* |  | Allograft replacement of coronary artery |
| K43* |  | Prosthetic replacement of coronary artery |
| K44* |  | Other replacement of coronary artery |
| K45* |  | Connection of thoracic artery to coronary artery |
| K46* |  | Other bypass of coronary artery |
| K49* |  | Transluminal balloon angioplasty of coronary artery |
| K50* |  | Other therapeutic transluminal operations on coronary artery |
| K75* |  | Percutaneous transluminal balloon angioplasty and insertion of stent into coronary artery |

* Codes used to identify both incident and prevalent CHD (the remaining codes were used to identify prior CHD at baseline only). ICD-9 codes were never used to identify incident CHD in the dataset).

# **Figure S1 Flowchart of exclusion criteria for the study population**

**Participants with baseline NMR-metabolomics profiling in UK Biobank: N=117,980**

**Participants included in main prospective analysis: N=89,242**

**(3,815 incident CHD)**

**Participants eligible for inclusion: N=117,938**

**Exclusion of participants use statin at baseline: N=19,022**

**Exclusion of participants with coronary heart disease at baseline: N=6,433**

**Exclusion of participants with missing/outlying values*****: N=3,241**

Missing values of exposure (n=82)

Missing values of covariates (n=2587)

Outliers in exposure (n=49)

Outliers in covariates (n=541)

Outliers in covariates (n=68)

**Exclusion of participants with duplicate samples, technical errors or missing data for all metabolic biomarkers: N=37**

**Exclusion of participants who withdrew from the cohort: N=5**

*Outliers of exposure are the metabolites outside the range of 4 standard deviation and out of 0.003% of either side; outliers of covariates are the participants less than 40, or equal or larger than 70 years old.

# **Table S2 Major components of incident coronary heart disease**

| **Components** | **ICD-10/OPCS codes** | **Number of events** |
| --- | --- | --- |
| CHD Death | I20-I25 from ONS | 488 |
| Unstable Angina | I20.0 from HES | 47 |
| Myocardial Infarction | I21.0-I21.4, I21.9, I22.0, I22.1, I22.8, I22.9, I23.0-I23.6, I23.8, I24.1, I24.8, I24.9, I25.1, I25.2, I25.5, I25.6, I25.8, I25.9 from HES | 3,235 |
| CHD-related Operation | K40-K45 from HES | 45 |
| **Total CHD** |  | 3,815 |

# **Table S3 Baseline medications and dietary habits by incident coronary heart disease**

|  | **Incident coronary heart disease** | |  |
| --- | --- | --- | --- |
|  | **No** | **Yes** | **All** |
|  |  |  |  |
| **No. of participants** | 85,427 | 3,815 | 89,242 |
|  |  |  |  |
| **Treatment and medications** | |  |  |
| Hypertension treatment, % | 12.1 | 22.8 | 12.5 |
| Atypical antipsychotic medication, % | 0.2 | 0.3 | 0.2 |
| Regular steroid tablets, % | 0.7 | 1.9 | 0.8 |
|  |  |  |  |
| **Supplements** |  |  |  |
| Vitamin supplement, % (missing=326) | 31.1 | 29.3 | 30.9 |
| Mineral supplement, % (missing=158) | 22.6 | 20.2 | 22.5 |
| Fish oil, % (missing=158) | 28.0 | 30.5 | 28.1 |
| Glucosamine, % (missing=158) | 7.3 | 6.8 | 7.3 |
|  |  |  |  |
| **Fresh fruit** (missing=0) |  |  |  |
| < 1 serves/day* | 9.6 | 13.1 | 9.7 |
| 1-<2 serves/day | 26.5 | 28.3 | 26.6 |
| 2-<3 serves/day | 28.3 | 26.6 | 28.3 |
| ≥ 3 serves/day | 35.6 | 32.0 | 35.5 |
|  |  |  |  |
| **Vegetables** (missing=0) | |  |  |
| <1 serves/day**^†^** | 10.5 | 13.8 | 10.6 |
| 1-<2 serves/day | 26.0 | 25.9 | 26.0 |
| 2-<3 serves/day | 33.2 | 31.1 | 33.2 |
| ≥ 3 serves/day | 30.3 | 29.2 | 30.2 |
|  |  |  |  |
| **Red meat** (missing=268) | |  |  |
| 0 times/week | 7.4 | 5.3 | 7.3 |
| 1 times/week | 3.5 | 3.6 | 3.5 |
| ≥ 1 times/week | 88.9 | 90.7 | 88.9 |
|  |  |  |  |
| **Oily fish** (missing=467) | |  |  |
| 0 times/week | 11.2 | 11.7 | 11.2 |
| 1 times/week | 34.0 | 33.5 | 34.0 |
| ≥ 1 times/week | 54.3 | 54.1 | 54.3 |
|  |  |  |  |
| **Whole grains** (missing=218) | |  |  |
| 0 serves/day^ǂ^ | 19.8 | 23.9 | 20.0 |
| <1 serves/day | 18.3 | 18.0 | 18.3 |
| 1-<3 serves/day | 46.7 | 42.2 | 46.5 |
| ≥ 3 serves/day | 14.9 | 15.7 | 14.9 |
|  |  |  |  |
| **Cheese** (missing=1880) | |  |  |
| <1 times/week | 18.8 | 18.6 | 19.2 |
| 1 times/week | 20.2 | 22.8 | 20.4 |
| 2<5 times/week | 44.9 | 43.8 | 44.9 |
| ≥ 5 times/week | 14.0 | 12.0 | 13.9 |
|  |  |  |  |
| **No dairy products, %** | 2.1 | 2.8 | 2.1 |
|  |  |  |  |
| Baseline characteristics of those with and without incident coronary heart disease during follow up among 89,242 participants (exclusions as in Figure S1). Categorical variables are presented as column percentages. * One prune, or one dried apricot, or 10 raisins as one serve; **^†^** Two heaped tablespoons of vegetables of one serve; ^ǂ^ One slice of bread, or one bowl of cereal as one serve; | | | |

# **Table S4 Correlations of baseline fatty acids concentration**

| **Fatty acids** | **Total FA** | **SFA** | **MUFA** | **Omega-6 PUFA** | **LA** | **Omega-3 PUFA** | **DHA** |
| --- | --- | --- | --- | --- | --- | --- | --- |
|  |  |  |  |  |  |  |  |
| **Total FA** | **1** | 0.97 | 0.95 | 0.86 | 0.81 | 0.53 | 0.28 |
| **SFA** |  | **1** | 0.93 | 0.75 | 0.68 | 0.47 | 0.21 |
| **MUFA** |  |  | **1** | 0.73 | 0.69 | 0.38 | 0.07 |
| **Omega-6 PUFA** |  |  |  | **1** | 0.98 | 0.43 | 0.30 |
| **Linoleic acids** |  |  |  |  | **1** | 0.33 | 0.22 |
| **Omega-3 PUFA** |  |  |  |  |  | **1** | 0.91 |
| **DHA** |  |  |  |  |  |  | **1** |
|  |  |  |  |  |  |  |  |
| Spearman partial correlations of fatty acids concentration, adjusting for age and sex; exclusions as in Figure S1. FA=fatty acids; SFA=Saturated fatty acids; MUFA=Monounsaturated fatty acids; PUFA=Polyunsaturated fatty acids; DHA= Docosahexaenoic acid. | | | | | | | |

# **Table S5 Correlations of baseline fatty acids concentration and lipid-related biomarkers**

| **Fatty acids** | **non-HDL-C** | **HDL-C** | **Total triglycerides** |
| --- | --- | --- | --- |
|  |  |  |  |
| **SFA** | 0.64 | 0.09 | 0.87 |
| **MUFA** | 0.57 | -0.11 | 0.93 |
| **PUFA** |  |  |  |
| **Omega-6 PUFA** | 0.82 | 0.26 | 0.59 |
| **Linoleic acids** | 0.79 | 0.20 | 0.58 |
| **Omega-3 PUFA** | 0.40 | 0.16 | 0.37 |
| **DHA** | 0.34 | 0.34 | 0.03 |
|  |  |  |  |
| Spearman partial correlations among fatty acids concentration or fatty acids ratio and lipid-related biomarkers, adjusting for age and sex. SFA=Saturated fatty acids; MUFA=Monounsaturated fatty acids; PUFA=Polyunsaturated fatty acids; DHA= Docosahexaenoic acid; LDL=Low-density lipoproteins; HDL=High-density lipoproteins; C=Cholesterol. | | | |

# **Table S****6 Baseline characteristics of the resurveyed population**

|  | **Female** | **Male** | **All** |
| --- | --- | --- | --- |
|  |  |  |  |
| **No. of participants** | 589 | 464 | 1,053 |
|  |  |  |  |
| **Age and socioeconomic factors** |  |  |  |
| Baseline Age (SD), years | 55.7 (7.3) | 56.8 (7.8) | 56.2 (7.6) |
| White, % | 97.5 | 98.3 | 97.8 |
| University education, % | 52.0 | 58.6 | 54.9 |
| Townsend deprivation index (SD) * | -2.0 (2.6) | -2.0 (2.8) | -2.0 (2.7) |
|  |  |  |  |
| **Lifestyle factors** |  |  |  |
| Current smoker, % | 3.2 | 6.7 | 4.7 |
| Current regular alcohol drinker, % | 68.6 | 82.3 | 74.6 |
|  |  |  |  |
| **Anthropometry** |  |  |  |
| Body Mass Index (SD), kg/m^2^ | 26.3 (4.7) | 26.9 (3.6) | 26.6 (4.3) |
| Waist circumference (SD), cm | 82.1 (11.1) | 94.0 (10.6) | 87.4 (12.4) |
| Waist to hip ratio (SD) | 0.81 (0.06) | 0.92 (0.07) | 0.86 (0.09) |
|  |  |  |  |
| **Lipids measured by clinical chemistry^†^** |  |  |  |
| LDL cholesterol (SD), mmol/l | 3.7 (0.8) | 3.6 (0.8) | 3.7 (0.8) |
| HDL cholesterol (SD), mmol/l | 1.6 (0.4) | 1.3 (0.3) | 1.5 (0.4) |
| Total triglycerides (SD), mmol/l | 1.5 (0.8) | 1.8 (1.0) | 1.6 (0.9) |
|  |  |  |  |
| **Blood pressure and diabetes** |  |  |  |
| Systolic blood pressure (SD), mmHg | 135.0 (17.9) | 140.8 (17.3) | 137.5 (17.9) |
| Diastolic blood pressure (SD), mmHg | 80.2 (9.2) | 83.7 (9.6) | 81.8 (9.5) |
| Baseline diabetes, % | 1.2 | 2.6 | 1.8 |
|  |  |  |  |
| **Fasting time (SD), h** | 3.6 (2.2) | 3.7 (2.5) | 3.6 (2.3) |
|  |  |  |  |
|  |  |  |  |

* Area-level measure of material deprivation, higher scores represent higher levels of deprivation

# **Table S7 Fatty acids biomarkers at baseline versus resurvey among the resurveyed population**

| **Biomarkers** | **Fatty acids concentration**  **(mmol/L)** | | **Fatty acids ratio (%),**  **relative to total fatty acids** | |
| --- | --- | --- | --- | --- |
|  | **Baseline**  **mean (SD)** | **Resurvey**  **mean (SD)** | **Baseline mean (SD)** | **Resurvey mean (SD)** |
|  |  |  |  |  |
| **SFA** | 4.1 (0.7) | 4.1 (0.9) | 34.0(1.2) | 33.9 (1.9) |
| **MUFA** | 2.8 (0.6) | 2.8 (0.8) | 23.3 (2.0) | 23.4 (2.3) |
| **PUFA** |  |  |  |  |
| **Omega-6 PUFA** | 4.5 (0.5) | 4.5 (0.7) | 38.4 (2.7) | 38.3 (3.2) |
| **Linoleic acids** | 3.5 (0.5) | 3.5 (0.7) | 29.6 (2.4) | 29.2 (3.1) |
| **Omega-3 PUFA** | 0.5 (0.2) | 0.5 (0.2) | 4.3 (1.2) | 4.4 (1.5) |
| **DHA** | 0.2 (0.1) | 0.2 (0.1) | 2.0 (0.5) | 2.0 (0.6) |
|  |  |  |  |  |
| SFA=Saturated fatty acids; MUFA=Monounsaturated fatty acids; PUFA=Polyunsaturated fatty acids; DHA= Docosahexaenoic acid. | | | | |

# **Table S8 Regression dilution ratios of fatty acids biomarkers**

| **RDR* (95%CI)** | **Fatty acids concentration**  **(mmol/L)** | **Fatty acids ratio (%),**  **relative to total fatty acids** |
| --- | --- | --- |
|  |  |  |
| **SFA** | 0.54 (0.49, 0.58) | 0.42 (0.37, 0.47) |
| **MUFA** | 0.58 (0.54, 0.62) | 0.62 (0.58, 0.66) |
| **PUFA** |  |  |
| **Omega-6 PUFA** | 0.54 (0.50, 0.58) | 0.59 (0.55, 0.63) |
| **Linoleic acids** | 0.52 (0.47, 0.56) | 0.56 (0.52, 0.60) |
| **Omega-3 PUFA** | 0.60 (0.56, 0.63) | 0.57 (0.53, 0.61) |
| **DHA** | 0.52 (0.47, 0.56) | 0.51 (0.46, 0.55) |
|  |  |  |
| *RDR=regression dilution ratio, estimated by the Spearman partial correlations between baseline and repeat measurements, adjusted for age groups and sex. SFA=Saturated fatty acids; MUFA=Monounsaturated fatty acids; PUFA=Polyunsaturated fatty acids; DHA= Docosahexaenoic acid. | | |

# **Table S9 Association of fatty acids concentration with coronary heart disease**

| Fatty acids | Fatty acids (mmol/L) | |  | A. Adjusted for age and sex | | |  | B. Further adjusted for social-economic and lifestyle factors | | |  | C. Further adjusted for body-mass index | | |
| --- | --- | --- | --- | --- | --- | --- | --- | --- | --- | --- | --- | --- | --- | --- |
|  |  |  |  |  |  |  |  |  |  |  |  |  |  |  |
|  | Baseline mean | Usual SD |  | HR (95% CI)* | LR χ^2^**^†^** | |  | HR (95% CI)* | LR χ^2^**^†^** | |  | HR (95% CI)* | LR χ^2^**^†^** | |
|  |  |  |  |  |  |  |  |  |  |  |  |  |  |  |
| **SFA** | 4.1 | 0.7 |  | 1.17 (1.14,1.21) | 107.9 |  |  | 1.16 (1.13,1.20) | 94.1 |  |  | 1.13 (1.09,1.16) | 58.4 |  |
| **MUFA** | 2.8 | 0.6 |  | 1.21 (1.18,1.24) | 159.2 |  |  | 1.18 (1.15,1.21) | 118.4 |  |  | 1.14 (1.11,1.18) | 71.7 |  |
| **PUFA** |  |  |  |  |  |  |  |  |  |  |  |  |  |  |
| **Omega-6 PUFA** | 4.5 | 0.5 |  | 1.12 (1.08,1.15) | 44.3 |  |  | 1.12 (1.09,1.16) | 49.0 |  |  | 1.11 (1.08,1.15) | 42.3 |  |
| **Linoleic acids** | 3.5 | 0.5 |  | 1.11 (1.07,1.14) | 39.8 |  |  | 1.12 (1.08,1.15) | 45.3 |  |  | 1.11 (1.08,1.15) | 42.7 |  |
| **Omega-3 PUFA** | 0.5 | 0.2 |  | 0.94 (0.91,0.98) | 11.3 |  |  | 0.99 (0.96,1.02) | 0.3 |  |  | 0.99 (0.95,1.02) | 0.6 |  |
| **DHA** | 0.2 | 0.1 |  | 0.87 (0.84,0.90) | 64.3 |  |  | 0.93 (0.90,0.96) | 18.8 |  |  | 0.94 (0.91,0.98) | 10.6 |  |
|  |  |  |  |  |  |  |  |  |  |  |  |  |  |  |
| Hazard ratios (HR) per usual SD higher level of mean fatty acids concentration among 89,242 participants (usual SD were estimated by 1,053 resurveyed participants). *HRs were calculated by Cox proportional-hazards models with: (A) stratification by age and sex; (B) stratification by age and sex, and adjusted for ethnicity, education, region, Townsend Deprivation Index, smoking, and alcohol; (C) model B with further adjustment for body-mass index. **^†^**Likelihood ratio (LR) χ2 improvement with the addition of the given factors to the model with stated adjustments. SFA=Saturated fatty acids; MUFA=Monounsaturated fatty acids; PUFA=Polyunsaturated fatty acids; DHA= Docosahexaenoic acid. | | | | | | | | | | | | | | |

# **Figure S2 Fatty acids concentration vs coronary heart disease risk in minimally adjusted model**


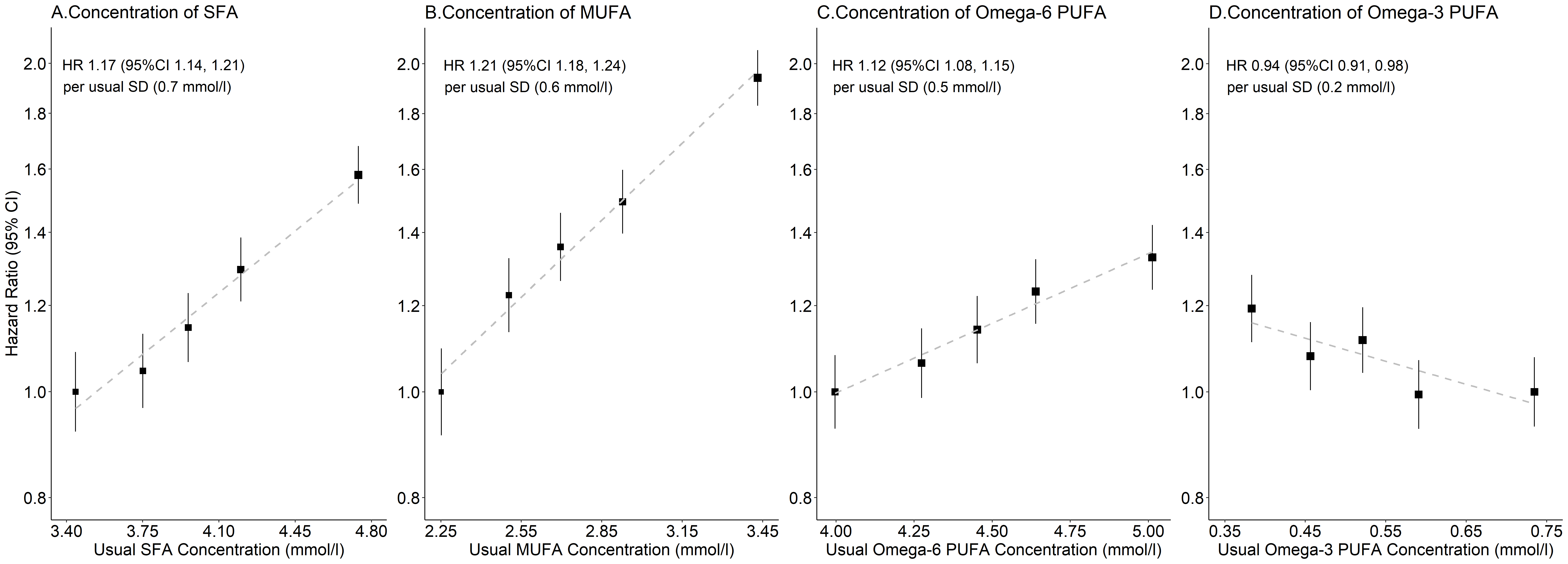


Hazard ratios (HR) per usual SD higher level of fatty acids concentration among 89,242 participants (exclusions as in Figure S1). HRs calculated by Cox proportional-hazards models, stratified by age and sex, without further adjustment. Area of the square is inversely proportional to the variance of the category-specific log risk. SFA= Saturated FA; MUFA= Monounsaturated fatty acids; PUFA=Polyunsaturated fatty acids.

# **Figure S3 Subtype of polyunsaturated fatty acids concentration vs coronary heart disease risk, given other fatty acids**


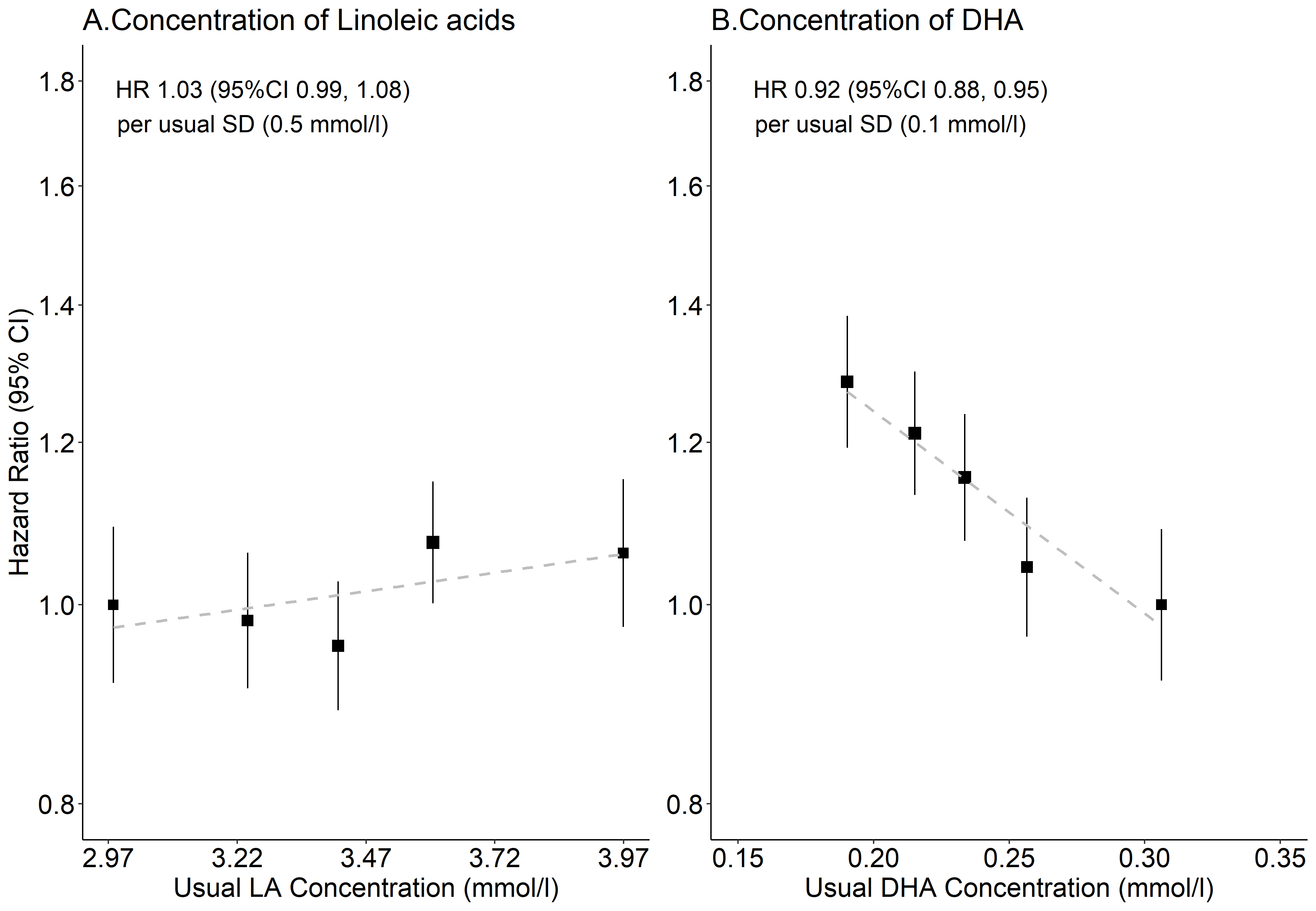


Hazard ratios (HR) per usual SD higher level of fatty acids concentration among 89,242 participants (exclusions as in Figure S1). HRs calculated by Cox proportional-hazards models, stratified by age and sex, and adjusted for ethnicity, education, region, Townsend Deprivation Index, smoking, alcohol, body-mass index, and mutual adjusted for other fatty acids (adjust for omega-3 FA, saturated FA and monounsaturated FA for the analysis of linoleic acids; adjust for omega-6 FA, saturated FA and monounsaturated FA for the analysis of DHA). Area of the square is inversely proportional to the variance of the category-specific log risk. DHA= Docosahexaenoic acid.

# **Table S10 Sensitivity analyses of fatty acids and coronary heart disease incidence**

| **Fatty acids biomarkers** | **Original model** | | |  | **Exclusion of events in first two years of follow-up** | | |  | **Further adjustment for other potential confounders** | | |  | **Further adjustment for blood pressure and diabetes** | | |
| --- | --- | --- | --- | --- | --- | --- | --- | --- | --- | --- | --- | --- | --- | --- | --- |
|  | HR (95% CI) | LR χ^2^ | |  | HR (95% CI) | LR χ^2^ | |  | HR (95% CI) | LR χ^2^ | |  | HR (95% CI) | LR χ^2^ | |
| **Concentration** |  |  |  |  |  |  |  |  |  |  |  |  |  |  |  |
| **SFA** | 1.14 (1.09, 1.20) | 31.1 |  |  | 1.14 (1.09, 1.20) | 28.5 |  |  | 1.16 (1.10, 1.22) | 31.1 |  |  | 1.13 (1.07, 1.19) | 19.5 |  |
| **MUFA** | 1.15 (1.10, 1.21) | 38.9 |  |  | 1.15 (1.10, 1.21) | 33.4 |  |  | 1.15 (1.10, 1.21) | 31.4 |  |  | 1.13 (1.08, 1.19) | 23.5 |  |
| **PUFA** |  |  |  |  |  |  |  |  |  |  |  |  |  |  |  |
| **Omega-6** | 1.04 (0.99, 1.09) | 2.2 |  |  | 1.05 (0.99, 1.10) | 3.0 |  |  | 1.05 (0.99, 1.10) | 2.7 |  |  | 1.05 (0.99, 1.10) | 3.0 |  |
| **Linoleic acids** | 1.03 (0.99, 1.08) | 1.9 |  |  | 1.04 (0.99, 1.09) | 2.4 |  |  | 1.04 (0.99, 1.09) | 2.4 |  |  | 1.05 (1.00, 1.10) | 3.3 |  |
| **Omega-3** | 0.91 (0.87, 0.94) | 23.5 |  |  | 0.91 (0.87, 0.95) | 21.3 |  |  | 0.90 (0.85, 0.94) | 21.0 |  |  | 0.90 (0.86, 0.95) | 17.4 |  |
| **DHA** | 0.92 (0.88, 0.95) | 19.9 |  |  | 0.92 (0.88, 0.96) | 17.9 |  |  | 0.91 (0.87, 0.95) | 16.6 |  |  | 0.91 (0.87, 0.96) | 14.5 |  |
|  |  |  |  |  |  |  |  |  |  |  |  |  |  |  |  |
| **Ratio to total FA** |  |  |  |  |  |  |  |  |  |  |  |  |  |  |  |
| **SFA** | 1.06 (1.03,1.09) | 12.7 |  |  | 1.07 (1.03, 1.10) | 13.8 |  |  | 1.08 (1.04, 1.11) | 16.7 |  |  | 1.05 (1.01, 1.09) | 7.5 |  |
| **MUFA** | 1.16 (1.12,1.20) | 76.3 |  |  | 1.16 (1.12, 1.20) | 64.7 |  |  | 1.15 (1.11, 1.20) | 51.9 |  |  | 1.14 (1.09, 1.18) | 42.1 |  |
| **PUFA** |  |  |  |  |  |  |  |  |  |  |  |  |  |  |  |
| **Omega-6** | 0.91 (0.88,0.94) | 35.1 |  |  | 0.90 (0.87, 0.94) | 32.3 |  |  | 0.90 (0.87, 0.93) | 31.6 |  |  | 0.92 (0.89, 0.95) | 20.0 |  |
| **Linoleic acids** | 0.96 (0.93,0.99) | 7.0 |  |  | 0.96 (0.93, 0.99) | 5.6 |  |  | 0.96 (0.92, 0.99) | 6.0 |  |  | 0.97 (0.94, 1.01) | 2.0 |  |
| **Omega-3** | 0.92 (0.89,0.95) | 26.5 |  |  | 0.91 (0.88, 0.95) | 23.0 |  |  | 0.91 (0.87, 0.95) | 18.3 |  |  | 0.92 (0.88, 0.96) | 16.1 |  |
| **DHA** | 0.88 (0.85,0.91) | 57.2 |  |  | 0.87 (0.84, 0.91) | 52.2 |  |  | 0.86 (0.83, 0.90) | 44.8 |  |  | 0.87 (0.84, 0.91) | 37.7 |  |
|  |  |  |  |  |  |  |  |  |  |  |  |  |  |  |  |
| Hazard ratios (HR) per usual SD higher level of fatty acids concentration (mmol/L) or ratio (%). HRs calculated by Cox proportional-hazards models, stratified by age and sex, ‘Original model’ is among 89,242 participants, with adjustment included ethnicity, education, region, Townsend Deprivation Index, smoking, alcohol, and body mass index, and models for concentration biomarkers were mutual adjusted for other fatty acids (as last row of each type of fatty acids in Figure 1) (same as the last columns of Table 2 and Table 3). ‘Further adjustments for other potential confounders' included further adjustment for waist circumference, fasting time, dietary factors (intake frequency of whole grains, fruit, vegetables, cheese, red meat, oily fish, and intake of dairy product) and spectrometer, among 86,164 participants with complete data. ‘Further adjustments for blood pressure and diabetes' included systolic blood pressure and diabetes at baseline, which might be mediators; ‘Exclusion of events in first two years of follow-up’ excluded 437 CHD events. Likelihood ratio (LR) χ^2^ improvement with the addition of the given factors to the model with stated adjustments. SFA=Saturated fatty acids; MUFA=Monounsaturated fatty acids; PUFA=Polyunsaturated fatty acids; DHA= Docosahexaenoic acid. | | | | | | | | | | | | | | | |

# **Figure S4 Fatty acids ratio vs coronary heart disease risk in fully adjusted model**


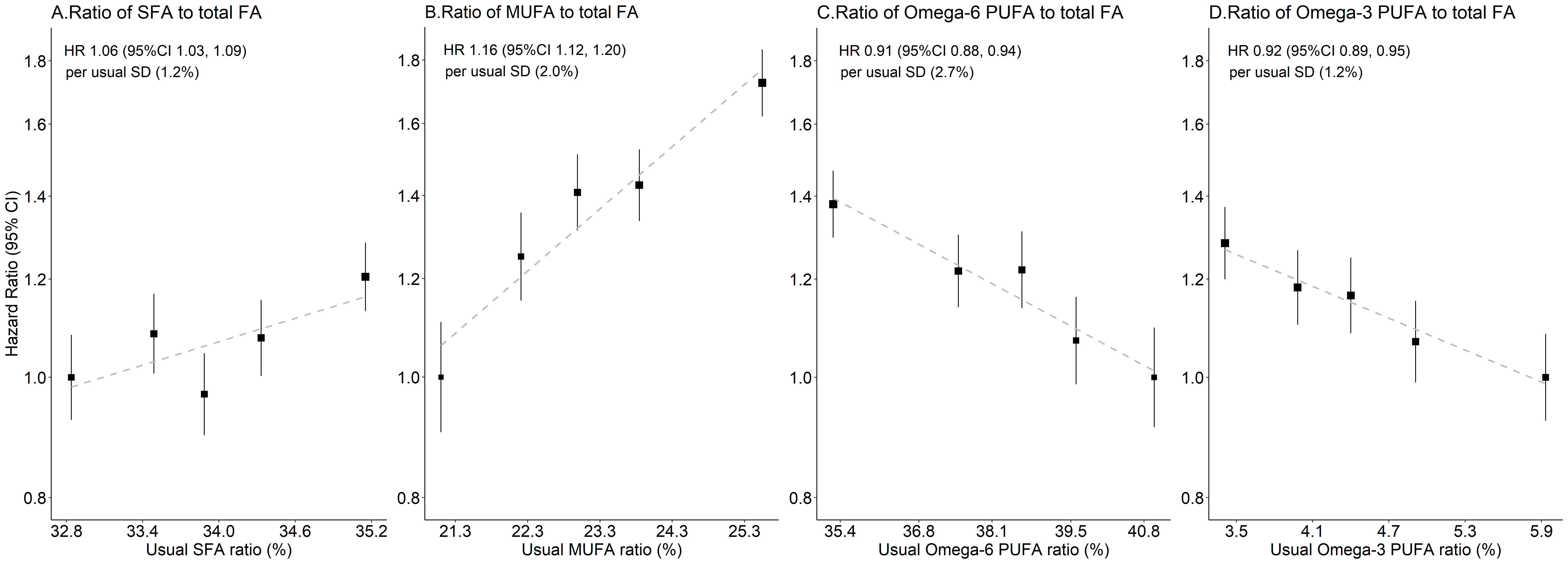


Hazard ratios (HR) per usual SD higher level of fatty acids ratio among 89,242 participants (exclusions as in Figure S1). HRs calculated by Cox proportional-hazards models, stratified by age and sex, and adjusted for ethnicity, education, region, Townsend Deprivation Index, smoking, alcohol, and body-mass index. Area of the square is inversely proportional to the variance of the category-specific log risk. SFA=Saturated fatty acids; MUFA=Monounsaturated fatty acids; PUFA=Polyunsaturated fatty acids.

# **Figure S5 Risk of coronary heart disease by usual fatty acids ratio, with further adjustment for lipids**

**
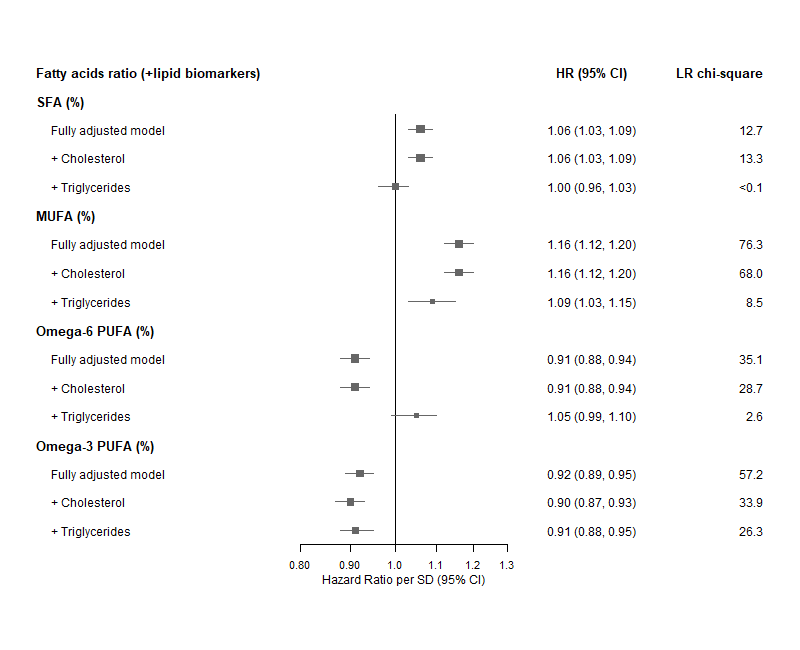
**

Hazard ratios (HR) per usual SD higher level of mean fatty acids ratio among 89,242 participants. The fully adjusted model represented Cox proportional-hazards model with stratification by age and sex, and adjustment for ethnicity, education, region, Townsend Deprivation Index, smoking, alcohol, and body-mass index (as last column of each type of fatty acids in Table 3), and further adjustment for non-HDL-cholesterol and HDL-cholesterol, or further adjustment for total triglycerides. Likelihood ratio (LR) χ2 improvement with the addition of the given factors to the model with stated adjustments. SFA=Saturated fatty acids; MUFA=Monounsaturated fatty acids; PUFA=Polyunsaturated fatty acids.

# **Figure S6 Fatty acids concentration vs coronary heart disease risk among participants taking versus not taking statin at baseline, given other fatty acids**


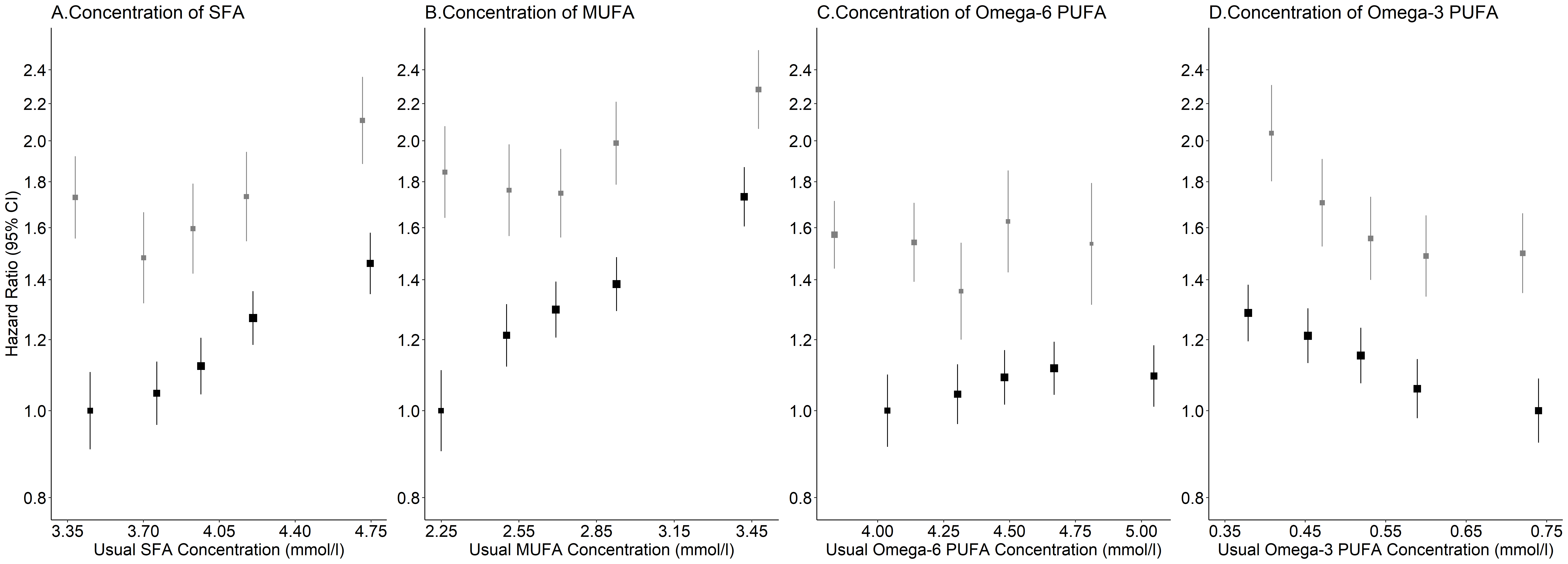


Hazard ratios (HR) per usual SD higher level of fatty acids concentration for baseline statin user (grey dots, 19,022 participants) and non-statin user (black dots, 89,242 participants). HRs are estimated by the stratified Cox proportional-hazards model by age and sex, and adjusted for ethnicity, education, region, Townsend Deprivation Index, smoking, alcohol, body-mass index, and mutual adjusted for other fatty acids (as last row of each type of fatty acids in Figure 1). Area of the square is inversely proportional to the variance of the category-specific log risk. SFA=Saturated fatty acids; MUFA=Monounsaturated fatty acids; PUFA=Polyunsaturated fatty acids.
